# Enhancing the functional properties of chitosan-alginate edible films using spent coffee ground extract for fresh-cut fruit preservation

**DOI:** 10.1016/j.crfs.2025.101124

**Published:** 2025-06-20

**Authors:** Oghenetega Lois Orhotohwo, Paolo Lucci, Amit K. Jaiswal, Swarna Jaiswal, Deborah Pacetti

**Affiliations:** a Department of Agricultural, Food and Environmental Sciences, Università Politecnica delle Marche, 60131, Ancona, Italy; b School of Food Science and Environmental Health, Faculty of Sciences and Health, Technological University Dublin - City Campus, Central Quad, Grangegorman, Dublin, Ireland; c Centre for Sustainable Packaging and Bioproducts, Technological University Dublin - City Campus, Central Quad, Grangegorman, Dublin, Ireland; d Sustainability and Health Research Hub, Technological University Dublin - City Campus, Grangegorman, Dublin, Ireland; e Health Engineering & Materials Science Research Hub, Technological University Dublin - City Campus, Grangegorman, Dublin, Ireland

**Keywords:** Edible films, Chitosan-alginate, Spent coffee ground extract, Active packaging, Food preservation

## Abstract

The developed edible film, formulated using sodium alginate, mushroom-derived chitosan, and bioactive-rich coffee ground extract, offers an environmentally friendly approach to food preservation. By utilizing agro-industrial byproducts, the formulation demonstrates potential economic and social benefits aligned with sustainable development goals. This study developed and optimised a sustainable edible film incorporating spent coffee ground extract (SCGE) as an active ingredient. The formulation was optimised through response surface methodology employing a central composite design, wherein chitosan (0.5–2.5 % w/v), sodium alginate (0.5–2.5 % w/v), glycerol (0.5–2.5 % v/v), and SCGE (4–12 % v/v) were systematically varied. The optimal film (1.49 % chitosan, 1.12 % sodium alginate, 1.53 % glycerol, and 9.69 % SCGE) exhibited enhanced mechanical strength (6.33 MPa), hydrophobicity (78.31° water contact angle), and antioxidant capacity. Spectroscopic analysis confirmed strong polymer–SCGE interactions, improving structural integrity and stability. Applying the coating to fresh-cut kiwi fruit notably decreased moisture loss by 37.5 %, delayed spoilage, and maintained appearance, and physicochemical characteristics over 10 days of cold storage at 4 °C. These results found SCGE-based edible films as an eco-friendly and functional packaging alternative, utilizing agro-industrial waste to enhance food quality, extend shelf life, and promote sustainability in contemporary food packaging.

## Highlights

• Chitosan-alginate films were optimised using the CCD design of RSM for enhanced functionality.
• SCGE incorporation increased the water contact angle to 79.13°, improving film hydrophobicity.
• The optimised film exhibited 87.52 % DPPH scavenging activity, enhancing antioxidant properties.
• SCGE-coated kiwifruit had 37.5 % less weight loss after 10 days of cold storage at 4 °C.
• SCGE-based coatings effectively delayed spoilage, supporting their use in active food packaging.

## Graphical abstract

**Figure:**
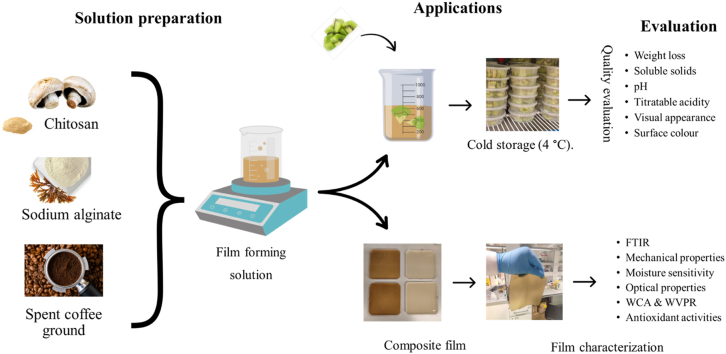

## 1 Introduction

Edible films and coatings represent innovative solutions to traditional plastic packaging, offering benefits such as biodegradability, functional versatility, and the potential to enhance the shelf life of food products. The materials, which consist of polysaccharides, proteins, or lipids, serve as carriers for natural antimicrobial and antioxidant agents, creating a protective barrier on food surfaces. By regulating moisture transfer, gas exchange, enzymatic activity, and microbial proliferation, edible films contribute to the preservation of food quality and the extension of storage life (Jurić et al., 2024; Karnwal et al., 2025). The physicochemical and functional properties of these films, such as mechanical strength, optical characteristics, and biodegradability, can be tailored by modifying the polymer composition, incorporating active ingredients, and optimising processing conditions, rendering them highly appropriate for various food uses (Gupta et al., 2024; Martins et al., 2024).

The food and beverage industry generates significant amounts of agro-industrial by-products, many of which contain bioactive compounds with functional properties. Coffee is one of the most frequently consumed beverages on a global scale, leading to substantial quantities of spent coffee grounds (SCG) being produced as waste. Approximately 6–8 million tonnes of SCG are generated annually, primarily originating from domestic brewing, commercial establishments, and the instant coffee industry. A significant portion of this organic waste is discarded, contributing to environmental pollution (Yusufoğlu et al., 2024).

However, SCG contains a range of bioactive compounds, including polyphenols, chlorogenic acids, caffeine, flavonoids, and melanoidins, which exhibit antioxidant, antimicrobial, and anti-inflammatory properties (Bevilacqua et al., 2023; Bouhzam et al., 2023). Despite these valuable properties, SCG remains underutilised, highlighting the need for innovative strategies to repurpose this by-product in high-value applications. Previous studies have investigated incorporating spent coffee ground extract (SCGE) into edible films, reporting notable enhancements in antioxidant activity and overall functional properties. For instance, SCGE-enriched polylactic acid (PLA)-based and whey protein films demonstrated significant radical scavenging capacity and improved structural integrity (Pettinato et al., 2023; Papadaki et al., 2022), reinforcing its potential in active packaging applications. These studies highlight SCGE's rich bioactive profile, excellent compatibility with various biopolymer matrices, thermal stability, and value as a repurposed agro-industrial byproduct. Compared to other natural additives, SCGE presents a cost-effective and environmentally sustainable alternative that aligns with circular economy principles while enhancing the functional performance of edible packaging materials.

Polysaccharides such as chitosan (Ch) and sodium alginate (SA) are widely employed as film-forming agents due to their biocompatibility, affordability, and excellent physicochemical properties (Hisham et al., 2024). Chitosan, a biopolymer derived from chitin, exhibits intrinsic antimicrobial activity, mechanical strength, and biodegradability, making it a suitable candidate for edible film production (Flórez et al., 2022). Sodium alginate, a naturally found anionic polymer derived from brown algae, offers excellent gel-forming and film-forming properties, as well as high flexibility and water solubility (Łętocha et al., 2022; Wang et al., 2024). When combined, chitosan and alginate form a polyelectrolyte complex (PEC) via electrostatic interactions between the protonated amine groups (-NH_3_^+^) of chitosan and the carboxylate groups (-COO^-^) of alginate. This interaction improves film stability, mechanical strength, and moisture resistance, making it highly suitable for food packaging applications (Xie et al., 2024). The incorporation of bioactive compounds, such as SCG extracts, into polysaccharide-based films leverages coffee waste's inherent antioxidant and photoprotective properties. This approach aligns with sustainable practices while enhancing the functional performance of food packaging materials—improving antioxidant activity, UV-blocking capacity, and barrier properties—thereby contributing to the development of active packaging systems (Bhattarai and Janaswamy, 2023). Beyond environmental benefits, edible packaging presents potential economic and social advantages. The use of mushroom processing waste (source for chitosan) and spent coffee grounds—as low-cost, readily available raw materials—can reduce production costs, making the technology more economically feasible, especially for small- and medium-scale enterprises. Moreover, this innovation supports circular economy principles by repurposing agro-industrial waste streams and adding value to otherwise discarded materials, potentially fostering local employment opportunities in biopolymer extraction and packaging production sectors (Dordevic et al., 2024; Lazaro-Molina and Lopez-Arenas, 2024; Pongsiriyakul et al., 2024).

The formulation of multifunctional edible films requires precise optimisation of biopolymer composition, plasticizer concentration, and active ingredient incorporation to achieve desirable physicochemical properties. Response Surface Methodology (RSM) is a statistical approach that enables the modelling and optimisation of complex formulations by evaluating the effects of multiple independent variables while reducing the number of experimental trials required (Mwelase et al., 2023). RSM has been successfully utilized to optimize edible coatings for a variety of fresh and minimally processed food products, including Orri mandarins (Soto-Muñoz et al., 2021), whole pomegranate fruit (Kawhena et al., 2021), tomato fruits (Tchouala Tao et al., 2023), and fresh date palm fruits (Mohammed et al., 2024). By employing a central composite design (CCD), this study systematically optimised the composition of SCGE-enriched chitosan-alginate films to achieve enhanced mechanical strength, water barrier properties, and antioxidant activity.

This study addresses existing gaps by developing, characterizing, and optimising edible films composed of chitosan and sodium alginate enriched with spent coffee ground extract (SCGE) using response surface methodology (RSM). While previous research has often focused on either polysaccharide-based films or the antioxidant potential of SCGE in isolation, this work explores their synergistic integration within a composite biopolymer matrix. The optimised films were evaluated for their effects on moisture retention, physicochemical stability, and shelf-life extension of fresh-cut kiwifruit during cold storage (4 °C). By combining principles of waste valorisation, sustainable packaging, and food preservation, this study contributes to the advancement of biodegradable active packaging solutions that address both food waste reduction and biopolymer functionality enhancement.

## 2 Materials and methods

### 2.1 Materials

Spent coffee ground was received from a local coffee shop in Ancona (Italy). They were oven-dried (Reciterm®, Isco Italy) at 50 °C until constant weight, vacuum-sealed and stored at room temperature. The mushroom, *Agaricus bisporus*, by-products for chitosan extraction were obtained from a local market in Dublin, Ireland. Food grade sodium alginate (Odaios Foods, Dublin, Ireland), glycerol (FCC, FG) from Sigma Aldrich (Ireland), and all other analytical grades of standards and chemicals used in this study were procured from Thermo Fisher Scientific, Dublin, Ireland. Kiwifruit used in the study were purchased from a local market in Dublin, Ireland.

### 2.2 Preparation of spent coffee ground extracts

The aqueous extract was prepared by combining the powder with distilled water (1:10, w/v) and subjecting it to heating in an ultrasonic bath (Elmasonic xtra ST, Elma Schmidbauer GmbH, Germany) at 55 °C for 30 min. The extracts were isolated from the solid phase using centrifugation (Sorvall ST 16 Centrifuge, Thermo Scientific) at 5000 rpm for 10 min at room temperature (20 ± 2 °C), and the supernatant was collected. The extraction was performed twice. The collected SCGE was preserved at −4 °C for subsequent utilization. The extraction yield was 16.0 %.

### 2.3 Extraction and characterisation of chitosan

According to Shahadha et al. (2023), the mushroom powder was combined with a 1M sodium hydroxide solution at a 1:30 (w/v) ratio and heated with moderate agitation at 100 °C for 3 h, with minor adjustments. The alkali-insoluble residue was rinsed with deionized water and centrifuged at 5000 rpm for 15 min at room temperature (20 ± 2 °C). The washing and centrifugation procedure was performed several times until a neutral pH was attained. The alkali-insoluble residue was subsequently freeze-dried using a Scanvac CoolSafe freeze dryer (LaboGene, Denmark). In the N-deacetylation phase, 1 g of crude chitin was subjected to a 50 % sodium hydroxide solution at a 1:50 (w/v) ratio and heated at 100 °C for 2 h. The product underwent washing with deionized water and was centrifuged at 5000 rpm for 15 min at room temperature (20 ± 2 °C), repeating this cycle until a neutral pH was attained. Chitosan was precipitated to produce chitosan powder through freeze-drying (Scanvac CoolSafe Freeze Dryer, LaboGene, Denmark).

The deacetylation degree (DD) of chitosan samples was assessed using the methodology outlined by Ssekatawa et al. (2021) by Fourier Transform Infrared Spectroscopy (FTIR). This method connects absorbance bands linked to specific amide, methyl, and hydroxyl groups detected in the FTIR spectra. The computation employed the Amide-I band at a wavenumber of 1655 cm^−1^ and the hydroxyl group band at 3450 cm^−1^, as shown in Equation (1):

(1) $$DD\;\left(\%\right)=100-\left[\frac{A_{1655}}{A_{3450}}\times \frac{100}{1.33}\right]$$

where A_1655_ represents the absorbance at 1655 cm^−1^ for the Amide-I band, indicating the content of N-acetyl groups. A_3450_ represents the absorbance at 3450 cm^−1^ for the hydroxyl band. The factor 1.33 corresponds to the ratio of A_1655_ to A_3450_ for fully N-acetylated chitosan.

### 2.4 Experimental design using response surface methodology

A central composite design (CCD) utilizing a quadratic model was employed as the design for the experiment. The experiment involved four independent variables: SCGE, chitosan, sodium alginate, and glycerol. Each independent variable was assessed at levels −1, 0, and +1. An entire set of 30 combinations consisting of six replicates of the centre point were selected in a random sequence based on a Central Composite Design (CCD) configuration for four factors, organized into three blocks. The formulations for the film/coating included chitosan at concentrations ranging from 0.5 % to 2.5 % w/v, sodium alginate at 0.5 %–2.5 % w/v, SCGE at 4 %–12 % v/v, and glycerol at 0.5 %–2.5 % v/v. The measured response function (y) included the edible film's thickness, tensile strength, and water contact angle.

The coefficient of determination (R^2^) was utilized to assess the fit quality for polynomial mathematical models. Optimisation was performed using numerical and graphical tools, incorporating four independent variables: the concentrations of SCGE, Ch, SA, and Gly. The optimisation procedures were conducted utilizing Design Expert Software Version 13, developed by Statease Inc. in MN, USA.

### 2.5 Development of film-forming solutions

The films were developed utilizing the methodology Bhatia et al. (2023) outlined, incorporating a few changes. Sodium alginate was prepared at concentrations ranging from 0.5 % to 2.5 % w/v, and chitosan was also prepared at concentrations between 0.5 % and 2.5 % w/v, with each component being processed separately. With continuous stirring, the chitosan was incrementally introduced into the sodium alginate solution at an equal volume ratio. Glycerol was incorporated as a plasticizer at a concentration ranging from 0.5 % to 2.5 % v/v. SCGE (4–12 % v/v) was incorporated into the film solution while stirring at 600 rpm and a temperature of 65 °C until a homogeneous mixture was achieved. Approximately 40 mL of film solution was dispensed into square petri dishes measuring 120 × 120 mm. The dishes were then maintained at ambient temperature for 48–72 h, allowing the solution to dry completely and detach from the surfaces of the petri dishes. The film was conditioned for 48 h at a temperature of 25 °C and a relative humidity of 50 % before further analysis. The amounts of sodium alginate, chitosan, glycerol, and SCGE used in the preparations of each formulation were selected according to Table 1. The selected range was based on preliminary lab studies.

**Table 1:** Central Composite Design (CCD) was employed for the formulation of a chitosan/alginate-based composite film incorporating spent coffee ground.

		Factor 1	Factor 2	Factor 3	Factor 4	Response 1	Response 2	Response 3
Run	Space Type	A:SCGE (% v/v)	B:Sodium Alginate (% w/v)	C:Chitosan (% w/v)	D:Glycerol (% v/v)	Thickness (mm)	Tensile strength (MPa)	WCA (°)
1	Axial	8	1.5	2.5	1.5	0.102	8.57	72.13
2	Factorial	6	2	1	2	0.111	5.55	72.71
3	Factorial	6	1	1	1	0.069	4.92	68.59
4	Centre	8	1.5	1.5	1.5	0.091	8.49	69.05
5	Centre	8	1.5	1.5	1.5	0.086	8.51	67.85
6	Factorial	6	2	2	1	0.084	17.32	61.03
7	Axial	8	0.5	1.5	1.5	0.063	1.77	85.44
8	Factorial	10	1	2	2	0.085	4.1	84.33
9	Axial	8	1.5	1.5	0.5	0.059	19.9	68.6
10	Factorial	6	1	2	2	0.086	4.01	78.46
11	Factorial	10	2	2	1	0.082	22.21	70.86
12	Centre	8	1.5	1.5	1.5	0.084	11.17	73.27
13	Factorial	10	2	1	1	0.067	16.55	56.67
14	Factorial	10	2	1	2	0.087	9.81	60.37
15	Factorial	10	1	1	1	0.056	7.92	74.09
16	Centre	8	1.5	1.5	1.5	0.081	7.06	74.94
17	Centre	8	1.5	1.5	1.5	0.079	8.92	71.91
18	Factorial	6	2	1	1	0.069	14.25	62.89
19	Factorial	6	2	2	2	0.099	11.13	69.71
20	Axial	8	1.5	0.5	1.5	0.081	9.35	76.1
21	Axial	8	2.5	1.5	1.5	0.098	15.64	59.02
22	Centre	8	1.5	1.5	1.5	0.091	7.68	73.77
23	Factorial	10	2	2	2	0.104	7.85	71.05
24	Factorial	10	1	2	1	0.07	9.37	74.83
25	Factorial	10	1	1	2	0.081	2.42	83.23
26	Axial	8	1.5	1.5	2.5	0.108	3.94	81.76
27	Axial	12	1.5	1.5	1.5	0.089	5.59	74.29
28	Factorial	6	1	1	2	0.077	2.39	83.65
29	Factorial	6	1	2	1	0.07	6.69	72.51
30	Axial	4	1.5	1.5	1.5	0.08	6.69	68.87

#### 2.5.1 Characterisation of response variables

##### 2.5.1.1 Determination of film thickness

ASTM D6988 is a widely acknowledged standard set by ASTM International for quantifying the thickness of the formed film. A digital micrometer (VWR, Ireland) with an accuracy of 0.001 mm was employed to measure the film thickness. The micrometer was randomly sampled from ten distinct positions for each film sample, and the mean value was computed.

##### 2.5.1.2 Tensile strength of the films

The mechanical strength of the packaging was assessed using the Standard ASTM D 882–88 method, conducted by an Instron Universal Testing Machine (Model 5565, Instron Engineering Corporation, Canton, MA, USA). The films were sectioned into strips measuring 3 × 10 cm. The experiment involved a grip length of 80 mm and a crosshead speed of 50 mm/min, utilizing a 500 N load cell on an Instron instrument. The test was conducted at room temperature until the sample failed at a specified location. The film's flexibility and strength were assessed through tensile properties, including tensile strength (TS), elongation at break (EaB), and elastic modulus (EM), as outlined by Perera et al. (2022). Only the TS was considered for the RSM optimisation of the composite films, and all three tensile properties in the characterisation of optimised film.

##### 2.5.1.3 Water contact angle (WCA)

The sessile drop technique was employed to assess the surface hydrophobicity of the films. The water contact angle was measured to assess the film's surface wettability, which refers to the interaction between the film surface and the liquid interphase. It evaluates the hydrophobic or hydrophilic properties of the surface. The static contact angle (θ) was determined utilizing water with an OCA Optical Tensiometer (DataPhysics Instruments GmbH, Filderstadt, Germany). The film specimen (8 × 8 cm) was placed on a stainless-steel pedestal. A microsyringe dispensed approximately 10 μL of distilled water onto the film's surface. The droplet's interaction on the film's surface was recorded with a high-speed camera and analyzed using the dpiMAX software from the OCA series. The measurement was based on the contact angle between the substrate surface and a tangent drawn from the edge to the droplet's contour. This was done on 10 different spots on the film samples and analyzed in triplicate.

### 2.6 Characterization of the optimised film composition

The optimal composition of the film (Ch_SA_SCGE) was characterised by structural, physicochemical, optical, mechanical, and active properties. A sample film of Ch_SA (excluding SCGE) was prepared and analyzed for a control experiment.

#### 2.6.1 Chemical structural and mechanical properties

The Fourier transform infrared (FT-IR) spectra of the films and the extract (SCGE, SA, Ch, Ch_SA, and Ch_SA_SCGE) were obtained using an FTIR spectrometer (Thermo Scientific, Ireland) within a spectral range of 4000-400 cm^−1^, at a resolution of 4 cm^−1^, with 64 scans (Augusto et al., 2018). The films' mechanical properties were assessed using the methodology outlined in Section 2.5.1.

#### 2.6.2 Moisture content and solubility

The moisture content and solubility of the optimised films were assessed following the methodology outlined by Lim et al. (2021). The film samples were prepared by cutting them into 2 × 2 cm dimensions, and the initial weight (W0) was meticulously recorded. The sample squares underwent drying in a Memmert UN55 oven at 60 °C for 24 h until a constant weight (W1) was achieved. The calculation of moisture content (%) was conducted using Equation (2):

(2) $$Moisture\;content\;\left(\%\right)=\frac{W0-W1}{W0}\times 100$$

Where W0 and W1 stand for the initial and final weight of the film, respectively.

Following the assessment of moisture content, the dried film samples (W1) were immersed and agitated in 25 mL of distilled water for 6 h at room temperature. The film samples underwent filtration prior to drying at 60 °C for 24 h in the oven. The final weight (W2) of the dried film was recorded. The solubility of films was determined using the subsequent Equation (3):

(3) $$Solubility\;\left(\%\right)=\frac{W1-W2}{W1}\times 100$$

where W1 and W2 stand for the weight of the initial and final dried film, respectively.

#### 2.6.3 Optical properties

The colour measurement of the film surface was conducted using an X-Rite 962/964 Spectrophotometer, employing a white colour sheet (L = 94.54, a = −1.11, b = −0.54) as the standard baseline. The color profile was defined using the Lab∗ color space, where L∗ ranges from 100 (white) to 0 (black), a∗ spans from negative values (indicating greenness) to positive values (indicating redness), and b∗ extends from negative values (representing blueness) to positive values (representing yellowness). The equation below computes the color difference (Shankar and Rhim, 2018). Ten measurements from three distinct replicates were documented, and the mean was calculated using Equation (4):

(4) $$\Delta E=\sqrt{{\left(\Delta L*\right)}^{2}+{\left(\Delta a*\right)}^{2}+{\left(\Delta b*\right)}^{2}}$$

where ΔL∗, Δa∗ and Δb∗ are changes in the values of colour parameters.

The opacity of the film samples was assessed using the methodology outlined by Zavareze et al. (2013), with the same equipment utilized for color measurements. The opacity (Y) of the samples was determined by the ratio of each sample's opacity on the black standard (Yb) to that on the white standard (Yw). The same equipment automatically made this calculation, Equation (5):

(5) $$Y=\frac{Y_{b}}{Y_{w}}$$

#### 2.6.4 Water contact angle and water vapour permeability rate

The same method as described in section 2.5.1 was used for determining the WCA of the optimised film samples. The film's water vapour permeability rate (WVPR) was assessed gravimetrically according to the method established by Perera et al. (2022). Fifteen grams of oven-dried CaCl_2_ were placed in a circular container with a diameter of 20 mm, utilizing a Memmert UN55 oven. The evaluated films (n = 3) covered the container's upper surface. The uncovered containers containing CaCl_2_ were designated as controls. The containers were positioned in the incubator at 25 °C and 95 % relative humidity for four days, with their weight recorded at 12-h intervals. The increased weight of the containers influenced the quantity of water vapour that permeated the film. The WVPR was determined using the following Equation (6):

(6) $$WVPR\left(g/{h.m}^{2}\right)=\frac{W1-W0}{A\times t}$$

where W0 is the initial film (g), W1 is the final film weight (g), at a time “t” in h and “A” represents the film's surface area (m^2^).

#### 2.6.5 Antioxidant activities of composite films

Extraction was conducted by dissolving 1 g of various film samples in 50 mL of water and stirring gently for 24 h at an ambient temperature to produce an edible film-soaking solution. The total phenolic content (TPC) of the produced films and the antioxidant capabilities were analyzed: DPPH radical scavenging assay and ABTS radical scavenging assay.

##### 2.6.5.1 Total phenolic content (TPC)

The TPC was determined according to Dou et al. (2018), with slight modifications. 1 mL of the extract solution was combined with 2.5 mL of the Folin-Ciocalteu reagent (10 % v/v), followed by adding 2 mL of sodium carbonate solution (7.5 % w/v) to the combination. The heterogeneous solution was maintained at ambient temperature for 30 min without light. The absorbance of the fluid was measured at 765 nm using a Thermo Scientific Multiskan GO Microplate Reader with SkanIt™ Software. The findings were reported in milligrams of gallic acid equivalents per gram of film (mg GAE/g film).

##### 2.6.5.2 DPPH radical scavenging assay

The in vitro antioxidant activity of the films was assessed using the 2,2-Diphenyl-1-picrylhydrazyl (DPPH) reduction technique, following the protocol established by Zheng et al. (2018), with minor changes. 3.0 mL of several edible film soaking solutions were combined with 3.0 mL of a 0.1 mM DPPH methanol solution. The mixture was agitated vigorously and maintained at ambient temperature in the absence of light for 30 min, after which the absorbance at 517 nm was quantified utilizing a Thermo Scientific Multiskan GO Microplate Reader with SkanIt™ Software. The activity was determined utilizing the following Equation (7):

(7) $$DPPH\;scavenging\;effect\;\left(\%\right)=\frac{{Abs}_{DPPH}-{Abs}_{extract}}{{Abs}_{DPPH}}\times 100$$

Where Abs_DPPH_ is the absorbance value at 517 nm of the methanolic solution of DPPH, and Abs_extract_ is the absorbance value at 517 nm for the sample extracts.

##### 2.6.5.3 ABTS radical scavenging assay

The ABTS radical scavenging activity assay was conducted following the methodology outlined by Hanani et al. (2019), with a few modifications. The stock solutions were prepared with 7.0 mM ABTS (2,2-azino-bis-3-ethylbenzothiazoline-6-sulphonic acid) solution and 2.45 mM potassium persulfate (K_2_S_2_O_8_) solution. The two stock solutions were combined in a 1:1 ratio and incubated at room temperature (20 ± 2 °C) for 16 h in the dark. The ABTS solution was diluted with ethanol until the initial absorbance reading of 0.70 ± 0.02 at 734 rpm was recorded using a Thermo Scientific Multiskan GO Microplate Spectrophotometer (Thermo Fisher Scientific, Ireland) with SkanIt™ software. The extract dilution and the ABTS solution (0.1:3, v/v) were combined, and the solution was incubated at room temperature for 6 min in the dark. The absorbance was determined at 734 nm. The extract was substituted with absolute ethanol to generate control. The subsequent Equation (8) was employed to determine the percentage of radical scavenging:

(8) $$ABTS\;scavenging\;effect\;\left(\%\right)=\frac{{Abs}_{control}-{Abs}_{sample}}{{Abs}_{control}}\times 100$$

where Abs_control_ is the absorbance of the control, and Abs_sample_ is the absorbance of the test sample. All tests were performed in triplicate.

### 2.7 Application and effect of the composite coatings on the quality of cut kiwifruit

The optimised film-forming solution, developed through response surface methodology (RSM), was applied as an edible coating on fresh-cut kiwifruit slices to assess its preservation performance. This approach was selected over pre-formed films to reflect practical application scenarios, as the coating is more suitable for fresh-cut produce. It ensures uniform surface coverage and better adherence, allowing a realistic evaluation of the film's effectiveness under storage conditions. The fruits were peeled manually and cut into cubes (approximately 2x2x2 cm). Fresh-cut cubes were dipped in the developed coating solution for 60 s. The excess coating was drained on a rack, and dried at ambient temperature for 20–30 min. The coating consisted of chitosan _sodium alginate (Ch_SA) and chitosan_sodium alginate_Spent coffee ground extracts (Ch_SA_SCGE). The control samples were kiwifruit cubes dipped in distilled water. Approximately 50 g of kiwifruit cubes were packed and sealed in polyethene terephthalate (PET) packages. They were stored at 4 ± 1 °C for 10 days. Physicochemical quality parameters were analyzed after 0, 2, 4, 6, 8, and 10 days of storage. The treated kiwifruit cubes were evaluated regarding the retention of physicochemical properties (total soluble solids, pH, and titratable acidity) and maintaining these properties throughout storage. Furthermore, the products were evaluated regarding weight loss and surface colour.

### 2.8 Statistical analysis

Experiments were carried out in triplicate for each experimental setup. The experimental design and data analysis were performed using Design Expert Software Version 13 (Statease Inc., MN, USA). A one-way analysis of variance (ANOVA) along with Fisher's least significant difference (LSD) test was employed to determine significant differences between the optimised film and other film samples, utilizing SPSS software (version 21.0, SPSS Inc., Chicago, IL, USA). The results are presented as mean ± standard deviation (SD), with a significance level of p < 0.05.

## 3 Results and discussion

### 3.1 Extraction and characterisation of chitosan

The undesirable portions of cultivated mushrooms were utilized for chitosan extraction, yielding 7.56 % chitosan. The extraction efficiency aligns with previously reported research but remains slightly lower than some reported values. Shahadha et al. (2023) obtained a 10.5 % yield from dried mushroom stems (*Agaricus bisporus*), while Wu et al. (2013) reported a lower yield of 5.74 %. Other studies have documented yields ranging from 5.81 % to 19.0 %, depending on the mushroom species and extraction methodologies (Chang et al., 2019; Ossamulu et al., 2023). The differences in chitosan yield reported across various studies can be influenced by factors such as the chitin content variation among mushroom species, disparities in cultivation conditions, and inconsistencies in extraction methods, particularly in the deproteinisation and deacetylation stages (Alimi et al., 2023).

Deacetylation, an essential process in converting chitin to chitosan, entails the elimination of acetyl groups, with the degree of deacetylation (DD) serving as a crucial factor influencing the physicochemical characteristics of chitosan. Chitosan is generally defined as the deacetylated form of chitin when the DD exceeds 50 % (Rinaudo, 2006). In this study, FTIR spectroscopy was employed to characterise the extracted chitosan, revealing a DD of 68.05 %, which confirms successful deacetylation. This value is comparable to those reported in the literature, including 66.35 % (Wu et al., 2013), 79 % (Periyannan et al., 2023), 83 % (Shahadha et al., 2023), and 69 % (Ozel and Elibol, 2024). The variation in DD observed across different studies can be influenced by processing conditions such as temperature, alkali concentration, reaction time, and the specific method of deacetylation used (Alimi et al., 2023). A higher degree of deacetylation enhances chitosan's solubility, bioactivity, and film-forming ability, making it a crucial parameter for applications in edible coatings and food packaging.

### 3.2 Optimisation of composite films using RSM

**Fig. 1:**
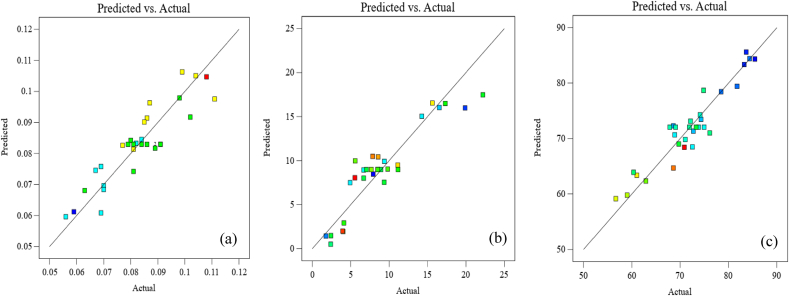
Correlations between predicted and experimental values of thickness (a), tensile strength (b), and water contact angle (c).

The effects of spent coffee ground extract (SCGE), chitosan (Ch), sodium alginate (SA), and glycerol (Gly) on the thickness, tensile strength (TS), and water contact angle (WCA) of the composite films were optimised using RSM with a Central Composite Design (CCD). A total of 30 experimental runs were conducted with different factor level combinations (Table 1 and S1). Statistical analysis, including ANOVA, regression coefficients (R^2^), and lack-of-fit tests, was performed (Table 2). The R^2^ values ranged from 0.8200 to 0.8893, indicating a strong correlation between the model predictions and experimental data. The difference between the adjusted R^2^ and predicted R^2^ was below 0.2, confirming the model's validity. Additionally, the model F-values were statistically significant (p < 0.05), and the lack-of-fit test was non-significant, suggesting the model was appropriate for explaining the variability in responses. Diagnostic plots (Fig. 1) showed a close alignment between predicted and actual values, further supporting the model's accuracy.

**Table 2:** Results of analysis of variance (ANOVA) and regression coefficients for all responses.

Source	Sum of Squares	df	Mean Square	F-value	p-value	
**Thickness (mm)**						

Model	0.0046	4	0.0012	28.48	<0.0001	significant
A-SCGE	9.375E-06	1	9.375E-06	0.2301	0.6357	
B-Sodium Alginate	0.0013	1	0.0013	32.76	<0.0001	
C-Chitosan	0.0005	1	0.0005	11.27	0.0025	
D-Glycerol	0.0028	1	0.0028	69.65	<0.0001	
Residual	0.0010	25	0.0000			
Lack of Fit	0.0009	20	0.0000	1.78	0.2715	not significant
Pure Error	0.0001	5	0.0000			
Cor Total	0.0057	29				
R^2^			0.8200			
Adjusted R^2^			0.7912			

**Tensile strength (MPa)**						

Model	653.43	4	163.36	33.69	<0.0001	significant
A-SCGE	5.77	1	5.77	1.19	0.2857	
B-Sodium Alginate	341.94	1	341.94	70.51	<0.0001	
C-Chitosan	12.48	1	12.48	2.57	0.1212	
D-Glycerol	293.23	1	293.23	60.47	<0.0001	
Residual	121.23	25	4.85			
Lack of Fit	111.30	20	5.56	2.80	0.1284	not significant
Pure Error	9.94	5	1.99			
Cor Total	774.66	29				
R^2^			0.8435			
Adjusted R^2^			0.8185			
						

**Water contact angle (°)**						

Model	1412.50	10	141.25	15.26	<0.0001	significant
A-SCGE	11.65	1	11.65	1.26	0.2760	
B-Sodium Alginate	903.32	1	903.32	97.57	<0.0001	
C-Chitosan	6.66	1	6.66	0.7191	0.4070	
D-Glycerol	325.31	1	325.31	35.14	<0.0001	
AB	26.68	1	26.68	2.88	0.1059	
AC	67.40	1	67.40	7.28	0.0142	
AD	18.02	1	18.02	1.95	0.1791	
BC	23.62	1	23.62	2.55	0.1267	
BD	18.62	1	18.62	2.01	0.1723	
CD	11.22	1	11.22	1.21	0.2847	
Residual	175.90	19	9.26			
Lack of Fit	136.83	14	9.77	1.25	0.4320	not significant
Pure Error	39.08	5	7.82			
Cor Total	1588.40	29				
R^2^			0.8893			
Adjusted R^2^			0.8310

A numerical response optimisation technique based on the desirability function was employed to determine the optimal composition of the chitosan–alginate edible film incorporating spent coffee ground extract (SCGE), specifically for high-moisture food applications. This multi-response optimisation approach simultaneously enhanced several key film properties within the experimentally observed parameter range.

The optimisation focused on four independent variables: SCGE (% v/v), chitosan (Ch, % w/v), sodium alginate (SA, % w/v), and glycerol (Gly, % w/v), targeting improvements in the film's physical, mechanical, and barrier characteristics. Response Surface Methodology (RSM) was utilized to model and optimize these variables. As predicted by the software, the optimal formulation was 9.7 % SCGE, 1.5 % chitosan, 1.1 % sodium alginate, and 1.5 % glycerol. The corresponding predicted response values were film thickness of 0.077 mm, tensile strength of 6.33 MPa, and a water contact angle (WCA) of 78.30°.

To validate the model, triplicate experiments were conducted using the predicted optimal formulation. The experimentally obtained values were 0.081 mm for thickness, 6.85 MPa for tensile strength, and 79.13° for WCA. The respective absolute residual errors were 4.94 %, 7.59 %, and 1.03 %, demonstrating a strong agreement between predicted and experimental results. These findings confirm the adequacy and reliability of the developed model and indicate that the optimised formulation is suitable for producing Ch_SA_SCGE-based edible films with favorable physical and mechanical properties, making it promising for application in sustainable food packaging.

### 3.3 Characterisation of the optimised film

#### 3.3.1 Chemical structural properties

**Fig. 2:**
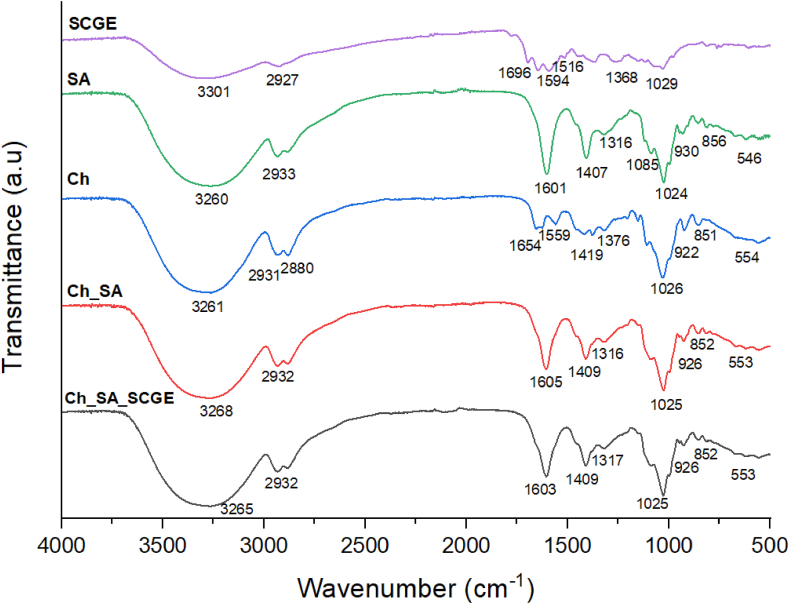
FTIR spectra of extract and film samples.

FTIR spectroscopy was conducted to analyse the intermolecular interactions between chitosan (Ch), sodium alginate (SA), and spent coffee ground extract (SCGE) in the optimised composite film (Fig. 2). Table S2 summarises the characteristic absorption bands of SCGE, SA, and Ch. The composite film spectrum exhibited peaks corresponding to both polymers, confirming the successful integration of SCGE into the Ch_SA matrix.

The incorporation of SCGE into the Ch_SA film induced notable spectral shifts, albeit of low magnitude. Specifically, the broad O–H and N–H stretching band at 3268 cm^−1^ and the C=O stretching band at 1605 cm^−1^ shifted slightly towards lower wavenumbers, with an increase in peak broadening. These shifts suggest specific molecular interactions, likely due to hydrogen bonding or electrostatic interactions between the functional groups of SCGE (e.g., hydroxyl, carboxyl, and phenolic groups) and the active sites within the Ch_SA matrix. Similar findings have been reported in gelatin-based films, where increasing SCGE concentration led to more pronounced intensity shifts, indicating stronger polymer-bioactive interactions (Getachew et al., 2021).

Comparable spectral modifications have also been recorded in chitosan films infused with plant extracts. For instance, the incorporation of tea extracts into chitosan films resulted in a shift of the typical C=O stretching and amide II bands at 1642 cm^−1^ and 1558 cm^−1^ to lower wavenumbers, which is attributed to interactions between the benzene rings of polyphenols and the chitosan matrix (Peng et al., 2013). These spectral shifts in the present study suggest good miscibility and structural compatibility between SCGE and the Ch_SA biopolymer matrix, reinforcing the enhanced functional properties observed in the optimised composite film.

#### 3.3.2 Film thickness and mechanical properties

Film thickness is a critical parameter in edible films, as it directly affects barrier properties, mechanical strength, and overall functionality (Nikoomanesh et al., 2024). An optimal thickness enhances the film's ability to prevent moisture loss and gas exchange, thereby extending shelf life while maintaining sufficient durability and consumer appeal. However, excessive thickness may compromise flexibility, increase material costs, and reduce transparency, making it essential to achieve a balance that maximises functionality, cost-efficiency, and sustainability in food packaging.

The findings indicated that the thickness of chitosan-only and alginate-only films did not differ significantly. However, the composite Ch_SA control film exhibited a significant increase in thickness, indicating that the combination of these two biopolymers resulted in a denser film structure. The addition of SCGE (Ch_SA_SCGE) led to a slight increase in thickness compared to the Ch_SA control film, although this change was not statistically significant (Table 3). These findings are consistent with Bhatia et al. (2023), who observed a similar trend in chitosan-sodium alginate edible films incorporated with ficus extract. Similarly, Kumar et al. (2021) reported that the integration of pomegranate peel extracts did not significantly alter the thickness of chitosan-based films, suggesting that the inclusion of plant extracts may modify film properties without substantially affecting structural dimensions.

**Table 3:** Thickness, moisture content, solubility, and mechanical properties of films.

Films	Thickness (mm)	Moisture content (%)	Solubility (%)	TS (MPa)	EaB (%)	YM (GPa)
**SA**	0.068 ± 0.004^b^	14.67 ± 0.44^b^	79.12 ± 1.39^a^	2.83 ± 0.19^c^	32.30 ± 0.49a	0.21 ± 0.02^c^
**Ch**	0.069 ± 0.005^b^	15.23 ± 0.17^b^	14.78 ± 0.58^d^	3.34 ± 0.35^c^	16.08 ± 1.39^c^	0.56 ± 0.02^a^
**Ch_SA**	0.079 ± 0.003^a^	17.98 ± 0.15^a^	20.89 ± 1.13^c^	4.50 ± 0.68^b^	27.51 ± 3.17^b^	0.45 ± 0.05^b^
**Ch_SA_SCGE**	0.081 ± 0.002^a^	14.48 ± 0.61^b^	43.28 ± 2.19^b^	6.85 ± 0.74^a^	36.19 ± 3.50^a^	0.53 ± 0.05^a^

Different letters in the same column indicate significant differences (*p* < 0.05). TS: tensile strength; EaB: elongation at break; YM: Young's modulus.

As shown in Table 3, the tensile strength (TS) of the Ch_SA control film was significantly lower than that of SCGE-enriched Ch_SA films. The incorporation of SCGE resulted in a notable increase in TS, indicating enhanced mechanical resistance. Additionally, a significant improvement was observed in elongation at break and Young's modulus, demonstrating that SCGE improved both film flexibility and elasticity. These results align with those of Getachew et al. (2021), who reported increased TS and Young's modulus in gelatin-based films with higher SCGE concentrations. Although the optimised film exhibited lower tensile strength than conventional plastic films (8–31 MPa) (Nilsen-Nygaard et al., 2021), its improved flexibility and reduced stiffness suggest it is more suitable for flexible packaging applications where high rigidity is not essential, such as single-use, short-shelf-life, or compostable packaging solutions.

Previous studies have shown that enriching edible films with bioactive extracts from agro-industrial by-products can improve their mechanical strength and flexibility due to enhanced molecular interactions within the polymer matrix. Vargas-Torrico et al. (2024) demonstrated that incorporating pomegranate peel extract into gelatin and carboxymethylcellulose-based films significantly increased elongation, puncture force, and TS values. Similarly, Dalei et al. (2024) found that the integration of cucumber peel extract into dragon fruit peel pectin-based films improved TS and Young's modulus. The enhancement in mechanical properties can be attributed to active compounds in plant extracts forming cross-links with polymer chains, thereby improving intermolecular cohesion and matrix distribution. This structural reinforcement results in a stronger, more elastic film, optimising durability and flexibility, both of which are essential for effective food packaging applications (Nastasi et al., 2023).

#### 3.3.3 Moisture content and water solubility

Moisture content and water solubility are essential factors in assessing the appropriateness of edible films for food packaging purposes. These characteristics impact film integrity, water resistance, and barrier efficacy, directly influencing packaged food items' shelf life and stability.

The Ch_SA control film exhibited the highest moisture content (17.98 %), which can be attributed to the hydrophilic nature of chitosan and sodium alginate and the formation of hydrogen-bonded networks within the polymer matrix. However, the incorporation of SCGE significantly reduced the moisture content by 19.47 % compared to the control film (Table 3). This reduction suggests that hydrophobic bioactive compounds present in SCGE interacted with the polymer matrix, reducing water affinity and retention. Such interactions likely altered the film's structural organisation, enhancing its barrier properties and decreasing water vapour permeability. Improved moisture resistance in edible films is beneficial for food packaging, as it limits moisture transfer, prevents microbial growth, and extends shelf life. These findings align with previous studies reporting similar reductions in moisture content upon the incorporation of bioactive extracts into edible films (Peng et al., 2013; Moghadam et al., 2020; Vargas-Torrico et al., 2022).

Water solubility is another crucial factor, particularly for biodegradable films, as it determines their resistance to humid environments and potential applications in water-sensitive food products. As shown in Table 3, the chitosan-only film exhibited the lowest solubility, consistent with chitosan's intrinsic water-resistant properties. However, the addition of sodium alginate increased film solubility, as alginate is more hydrophilic and promotes greater water absorption. The incorporation of SCGE further enhanced solubility, suggesting that the hydrophilic compounds present in the plant extract interacted with the film matrix, increasing its affinity for water and facilitating higher water absorption and dissolution. Similar effects were reported by Peng et al. (2013), where the addition of tea extracts significantly improved the solubility of chitosan films. While increased solubility may be beneficial for applications requiring rapid film degradation, it is essential to balance water resistance and biodegradability to ensure optimal functionality in food packaging.

The above characteristics are typical of bio-based films derived from natural polymers, which readily interact with water molecules due to hydrophilic functional groups such as hydroxyl and carboxyl groups. This interaction facilitates microbial degradation and promotes environmental disintegration, reinforcing the film's potential as a sustainable alternative to conventional plastics.

#### 3.3.4 Optical properties of composite films

The colour and opacity of edible films are crucial factors influencing consumer acceptance and product appearance. The L∗ (lightness), a∗ (red-green), b∗ (yellow-blue), total colour difference (ΔE), and opacity values of the films are presented in Table 4. The incorporation of SCGE resulted in a significant increase in a∗, b∗, and ΔE values compared to the Ch_SA control film, whereas L values decreased significantly∗. This change can be attributed to the dark brown pigmentation of SCGE, which alters the overall appearance of the composite film. Similar effects have been reported by Getachew et al. (2021), where the addition of SCGE in gelatin-based films caused increased a, b, and ΔE values∗∗ while decreasing L∗. Ballesteros et al. (2018) also observed comparable trends in carboxymethyl cellulose films containing spent coffee ground polysaccharides, further supporting this study's findings.

**Table 4:** Optical properties, water contact angle (WCA) and water vapour permeability rate (WVPR) of films.

Films	L∗	a∗	b∗	ΔE	Opacity (%)	WCA (°)	WVPR (g/h.m^2^)
**Control (no films)**	–	–	–	–	–	–	50.58 ± 2.13^b^
**SA**	91.16 ± 0.07^a^	−1.01 ± 1.34^c^	1.97 ± 0.24^d^	4.18 ± 0.34^d^	0.62 ± 0.06^d^	44.31 ± 2.89^c^	66.59 ± 0.34^a^
**Ch**	76.51 ± 1.36^c^	3.30 ± 0.55^b^	26.95 ± 1.34^b^	33.04 ± 1.90^b^	12.51 ± 0.91^a^	84.92 ± 2.31^a^	54.73 ± 0.10^b^
**Ch_SA**	84.91 ± 1.03^b^	−0.16 ± 0.24^c^	15.96 ± 1.23^c^	19.01 ± 1.56^c^	4.79 ± 0.79^c^	77.96 ± 3.74^b^	67.47 ± 1.63^a^
**Ch_SA_SCGE**	61.91 ± 1.06^d^	9.96 ± 0.57^a^	42.99 ± 0.32^a^	55.38 ± 0.81^a^	10.12 ± 0.65^b^	79.13 ± 1.93^b^	66.90 ± 1.10^a^

Different letters in the same column indicate significant differences (*p* < 0.05). L∗: lightness; a∗: red–green; b∗: yellow–blue; ΔE: colour difference.

Additionally, the chitosan-only film exhibited the highest opacity, which is likely due to the absence of a bleaching step during extraction. This resulted in a light brownish film due to the presence of residual pigments, contributing to increased opacity. Opacity plays a vital role in light protection, particularly for photosensitive food products, making such films suitable for applications where light exposure may accelerate quality deterioration.

#### 3.3.5 Water contact angle (WCA) and water vapour permeability rate (WVPR)

The wettability of the composite films was assessed by measuring the water contact angle (WCA), which indicates the hydrophilic or hydrophobic nature of the film surface. A lower WCA corresponds to higher surface wettability (hydrophilicity), whereas a higher WCA indicates an increased hydrophobic character. As presented in Table 4, the incorporation of SCGE into the Ch_SA film resulted in a slight increase in WCA, though the difference was not statistically significant. The observed increase suggests that phenolic compounds within SCGE may have influenced polymer interactions, altering surface properties. Similar findings were reported by Getachew et al. (2021), where increasing SCGE concentrations in fish skin gelatin-based films significantly increased WCA due to polymer–phenolic cross-linking, which modified the surface roughness and reduced water affinity.

Water vapour permeability rate (WVPR) is a key determinant of a film's moisture barrier effectiveness, directly impacting its suitability for food preservation applications (Yue et al., 2024). A lower WVPR indicates enhanced moisture resistance, which is essential for reducing water transfer, thereby preventing food spoilage and quality degradation (Giyatmi et al., 2021). As shown in Table 4, the WVPR of the films increased by 33.39 % and 32.27 % for Ch_SA and Ch_SA_SCGE, respectively, relative to the uncoated control sample. These findings indicate that SCGE slightly reduced WVPR, suggesting an improvement in moisture barrier properties. However, the difference between Ch_SA and Ch_SA_SCGE films was not statistically significant. Nilsen-Nygaard et al. (2021) reported that the water vapour permeability rate of bio-based films is significantly higher than that of conventional films. A similarly high WVPR was observed in our study, highlighting the inherent challenge and suggesting limited moisture resistance. Although, the slight reduction in WVPR may be attributed to the formation of a denser and more compact film structure, which limits moisture diffusion. This trend aligns with previous studies, where the incorporation of plant-based extracts into edible films led to reduced WVPR and enhanced barrier properties (Giyatmi et al., 2021), (Ali et al., 2023). Increasing SCGE concentrations in future formulations may further modulate the film's water resistance, improving its functionality as an active food packaging material.

#### 3.3.6 Antioxidant activities of composite films

The total phenolic content (TPC) of the dried spent coffee grounds (SCG) was determined using the Folin–Ciocalteu method and found to be 19.72 mg GAE/g of dry weight, indicating a substantial presence of antioxidant compounds. This notable level of phenolics suggests the suitability of SCG for incorporation into edible film formulations due to its potential to enhance antioxidant properties. Comparative literature reports a wide range of TPC values for SCG, from 15 to 34.43 mg GAE/g DW (Solomakou et al., 2022a) to considerably lower values of 8.0–8.4 mg GAE/g DW (Papadaki et al., 2022) and 6.8–18.0 mg GAE/g DW (Maiyah et al., 2025). Such variations can be attributed to differences in coffee bean variety, agricultural and environmental conditions (e.g., soil type, climate), as well as the extraction protocols employed (Solomakou et al., 2022a; Maiyah et al., 2025).

The relatively high TPC value observed in this study highlights the potential of SCG as a rich natural source of bioactive compounds. These phenolics, including chlorogenic acids and caffeine—both previously identified in SCG—are known for their antioxidant activity and have been shown to contribute to the functional performance of active packaging systems (Corrado et al., 2024). Notably, their presence likely contributed to the enhanced radical scavenging activity observed in the SCG extract (SCGE)-incorporated films developed in this work.

The incorporation of natural antioxidant agents into edible films has gained considerable interest due to their ability to delay oxidative degradation and extend the shelf life of food products (Singh et al., 2022). In this study, the total phenolic content (TPC) of the Ch_SA and Ch_SA_SCGE films was 0.06 mg GAE/g and 5.05 mg GAE/g, respectively, demonstrating a significant increase in polyphenolic compounds upon the incorporation of SCGE. The enhanced antioxidant activity observed in the Ch_SA_SCGE film is attributed to the rich polyphenolic profile of SCG, which includes chlorogenic acids, flavonoids, and other bioactive compounds known for their radical-scavenging properties (Okur et al., 2021; Solomakou et al., 2022b).

As illustrated in Fig. S1, the Ch_SA_SCGE film exhibited significantly higher scavenging activity for DPPH (87.52 %) and ABTS (64.84 %) radicals, compared to the Ch_SA control film, which showed lower inhibition levels of 39.11 % (DPPH) and 19.58 % (ABTS). These results indicate that SCGE-enriched films possess strong antioxidant potential, which could contribute to delaying lipid oxidation and preserving food quality. Similar findings were reported by Dordevic et al. (2024), where the addition of spent coffee emulsions to κ-carrageenan-based films resulted in a notable increase in TPC and antioxidant activity. Likewise, previous studies by Drago et al. (2022), Getachew et al. (2021), and Papadaki et al. (2022) confirmed that films enriched with spent coffee extract exhibited significantly higher antioxidant capacity, consistent with the current findings.

#### 3.3.7 Effect of coatings on the quality of fresh-cut kiwifruit

##### 3.3.7.1 Weight loss

**Fig. 3:**
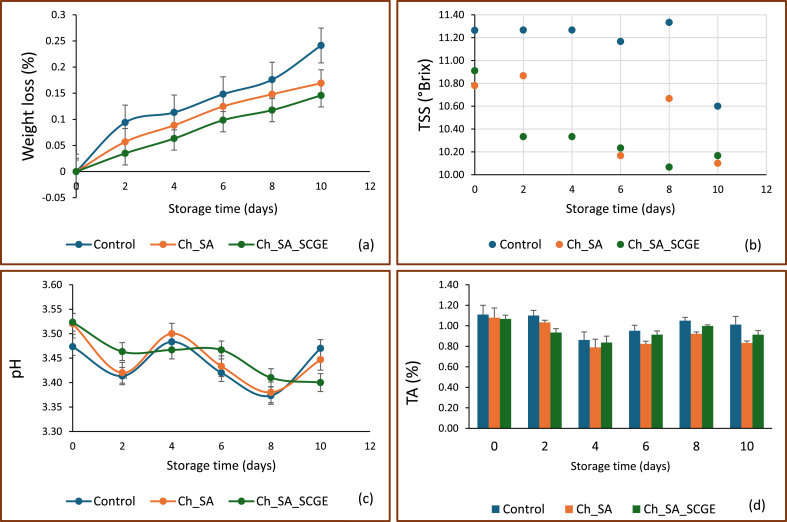
Change in weight loss (a), TSS (b), pH (c), and TA (d) of control and coated kiwifruit cubes during cold storage at 4 ± 1 °C for 10 days.

Fig. 3a illustrates the effect of optimised edible coatings on the weight loss of fresh-cut kiwifruit following a 10-day cold storage period at 4 °C. The Ch_SA and Ch_SA_SCGE coated fruits showed markedly reduced weight loss (0.17 % and 0.15 %, respectively) in contrast to the uncoated control, which saw a weight loss of 0.24 %. The results demonstrate that the edible coatings significantly minimized moisture loss, decreasing dehydration.

The reduction in weight loss can be attributed to the moisture barrier properties of the coatings, which limit water evaporation and transpiration rates. The slightly lower weight loss observed in Ch_SA_SCGE-coated fruits compared to Ch_SA-coated fruits suggests that the incorporation of SCGE may have enhanced the film structure, improving water retention within the fruit tissue. Similar trends have been reported in previous studies, where biopolymer-based edible coatings effectively reduced moisture loss and preserved the quality of fresh-cut fruits (Gullifa, 2021; Guroo et al., 2021; Manzoor et al., 2021; Sortino et al., 2022).

##### 3.3.7.2 Total soluble solids (TSS), pH and titratable acidity (TA)

Fig. 3(b–d) presents the impact of edible coating treatments on the chemical parameters of fresh-cut kiwifruit during cold storage at 4 °C. No significant variations in TSS were detected between treatments during the storage period, except for day 8, when slight variances were identified. Overall, TSS remained stable across all samples, with slight fluctuations. The control sample initially exhibited marginally higher TSS values, which were maintained throughout storage. These findings align with Hashemi & Jafarpour (Hashemi and Jafarpour, 2021), who reported a similar trend in fresh-cut kiwifruit coated with a konjac-based edible film containing *Lactobacillus plantarum*, where TSS values remained stable over five days of cold storage. Similarly, Benítez et al. (2013) found no significant changes in TSS in kiwifruit slices coated with aloe vera-based films, reinforcing the observation that edible coatings do not significantly alter soluble solids in fresh-cut fruit.

The pH values of all samples were similar at the beginning of the study but gradually declined over the storage period. A notable decrease in pH was observed in the control treatment by day 10, whereas the Ch_SA-coated samples exhibited only a minor decline over time. A gradual reduction in pH is typically associated with organic acid metabolism, enzymatic activity, and microbial growth during storage. The stability of pH in the coated samples suggests that the edible films may have contributed to maintaining biochemical stability, slowing the degradation process.

Regarding titratable acidity (TA), slight differences were observed among the samples throughout storage. By day 10, a more pronounced decline in TA was noted in Ch_SA-coated samples, whereas the Ch_SA_SCGE-coated fruits exhibited a relatively stable TA. The presence of SCGE in the coating may have contributed to this effect, potentially due to polyphenolic interactions with organic acids (Dordevic et al., 2024). These active coatings have been shown to maintain acidity during storage, likely by delaying the respiration process and thereby reducing the utilization of organic acids (Kaur et al., 2023).

##### 3.3.7.3 Colour

**Fig. 4:**
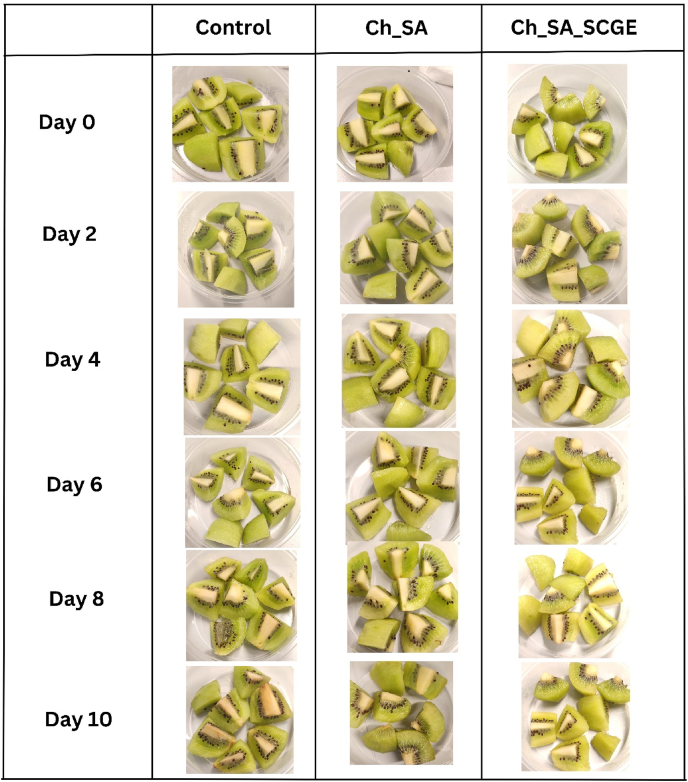
Visual appearance of coated and uncoated kiwifruit cubes during storage.

**Fig. 5:**
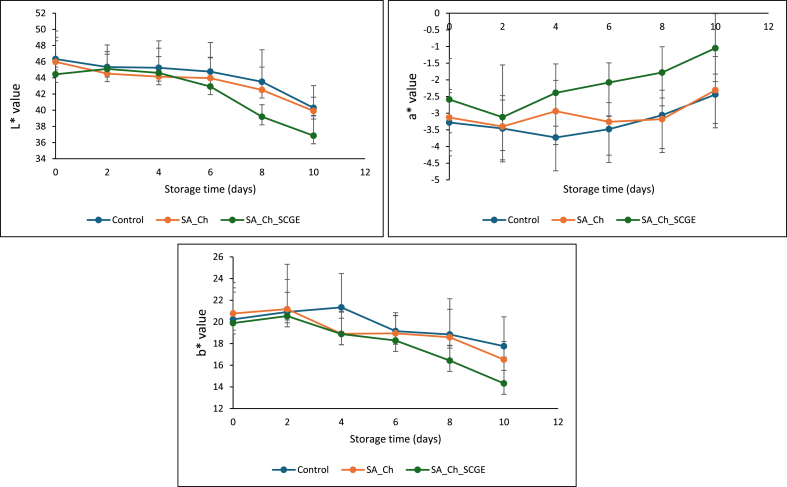
Colour changes in control and coated kiwifruit cubes during cold storage at 4 ± 1 °C for 10 days.

The appearance of coated and uncoated kiwifruit cubes during storage is shown in Fig. 4. By day 6, the uncoated control samples exhibited visible signs of deterioration, indicating spoilage, whereas the coated samples remained visually intact. Spoilage was evident in the Ch_SA-coated samples by day 10, while the Ch_SA_SCGE-coated samples maintained a better appearance, suggesting an extended shelf life due to the presence of SCGE. For the colour parameters (L∗, a∗, and b∗), all samples exhibited significant changes over time, indicating colour degradation (Fig. 5). A notable decrease in L (lightness)∗ was observed in SCGE-incorporated films, which may be attributed to interactions between SCGE polyphenols and kiwifruit pigments, leading to modifications in visual appearance. The incorporation of SCGE likely influenced oxidation and enzymatic browning reactions, altering colour intensity during storage. These findings are consistent with previous studies that reported significant reductions in brightness (L)∗ in kiwifruit slices treated with bioactive-enriched edible coatings under storage conditions (Sortino et al., 2022; Benítez et al., 2013). The observed changes highlight the importance of active coatings in maintaining fruit appearance, which is a crucial factor for consumer acceptance and marketability of fresh-cut produce.

While this application primarily focused on maintaining the quality and extending the shelf life of fresh-cut kiwifruit, it is important to note that microbial stability also significantly influences overall shelf life. As microbial parameters were not evaluated in the present work, future studies should consider incorporating microbial assessments to provide a more comprehensive understanding of the factors affecting the shelf life of fresh-cut fruits.

## 4 Conclusion

This study successfully developed and optimised chitosan-alginate edible films incorporating spent coffee ground extract as an active component using RSM. The optimised formulation, consisting of 1.49 % chitosan, 1.12 % sodium alginate, 1.53 % glycerol, and 9.69 % SCGE, exhibited enhanced physicochemical properties, including a tensile strength of 6.33 MPa, a thickness of 0.077 mm, and a water contact angle of 78.31°. The addition of SCGE markedly enhanced the antioxidant activity of the films, as evidenced by total phenolic content and radical scavenging experiments. Spectroscopic examination revealed significant intermolecular interactions between SCGE and the biopolymer matrix, enhancing structural integrity and functional stability. Applying the optimised edible coating to fresh-cut kiwifruit led to a substantial decrease in moisture loss, enhanced preservation of texture and colour, and prolonged shelf life during ten days of cold storage at 4 °C. The SCGE-enriched films demonstrated superior performance compared to uncoated samples, effectively delaying quality deterioration and maintaining physicochemical stability.

The biodegradable nature of these films, combined with their functional properties, underscores their promise as sustainable alternatives to conventional food packaging. This study demonstrates the potential of SCGE as a bioactive additive in edible films, offering the dual benefits of enhancing food preservation and promoting the valorisation of agro-industrial waste. While the results are encouraging, they are based on controlled laboratory conditions and a single film formulation, which may limit scalability and practical application. Further research should explore alternative film matrices and examine the migration behavior of phenolic compounds, as well as their interactions with food products, to better assess real-world performance.

## CRediT authorship contribution statement

**Oghenetega Lois Orhotohwo:** Conceptualization, Formal analysis, Data curation, Investigation, Methodology, Writing – original draft, Writing – review & editing. **Paolo Lucci:** Validation, Writing – review & editing. **Amit K. Jaiswal:** Conceptualization, Methodology, Resources, Supervision, Validation, Writing – review & editing. **Swarna Jaiswal:** Conceptualization, Methodology, Resources, Supervision, Validation, Writing – review & editing. **Deborah Pacetti:** Conceptualization, Project administration, Validation, Writing – review & editing.

## Declaration of competing interest

The authors declare that they have no known competing financial interests or personal relationships that could have appeared to influence the work reported in this paper.

## Data Availability

The datasets generated and/or analysed during the current study are available from the corresponding author upon reasonable request.
