# Supplementary material for: Enhancing the functional properties of chitosan-alginate edible films using spent coffee ground extract for fresh-cut fruit preservation
Source: Curr Res Food Sci. 2025 Jun 20;11:101124. doi: 10.1016/j.crfs.2025.101124 (PMC12226113; doi:10.1016/j.crfs.2025.101124)
Supplement: Multimedia component 1 [file mmc1.pdf]

Supplementary Material

Enhancing the functional properties of chitosan-alginate edible films using spent coffee ground extract for fresh-cut fruit preservation

Oghenetega Lois Orhotohwo*[a,b,c,d]*, Paolo Lucci*[a]*, Amit K. Jaiswal*[b,c,d,e]*, Swarna Jaiswal*[b,c,d,e], and Deborah Pacetti*[a]**

[a]Department of Agricultural, Food and Environmental Sciences, Università Politecnica delle Marche, 60131 Ancona, Italy.

[b]School of Food Science and Environmental Health, Faculty of Sciences and Health, Technological University Dublin - City Campus, Central Quad, Grangegorman, Dublin, Ireland

[c]Centre for Sustainable Packaging and Bioproducts, Technological University Dublin - City Campus, Central Quad, Grangegorman, Dublin, Ireland

[d]Sustainability and Health Research Hub, Technological University Dublin - City Campus, Grangegorman, Dublin, Ireland

[e]Health Engineering & Materials Science Research Hub, Technological University Dublin - City Campus, Grangegorman, Dublin, Ireland

*Corresponding author: Swarna Jaiswal

Email Id: swarna.jaiswal@tudublin.ie; swarna.jaiswal@outlook.com

ORCID ID: https://orcid.org/0000-0003-1414-9052

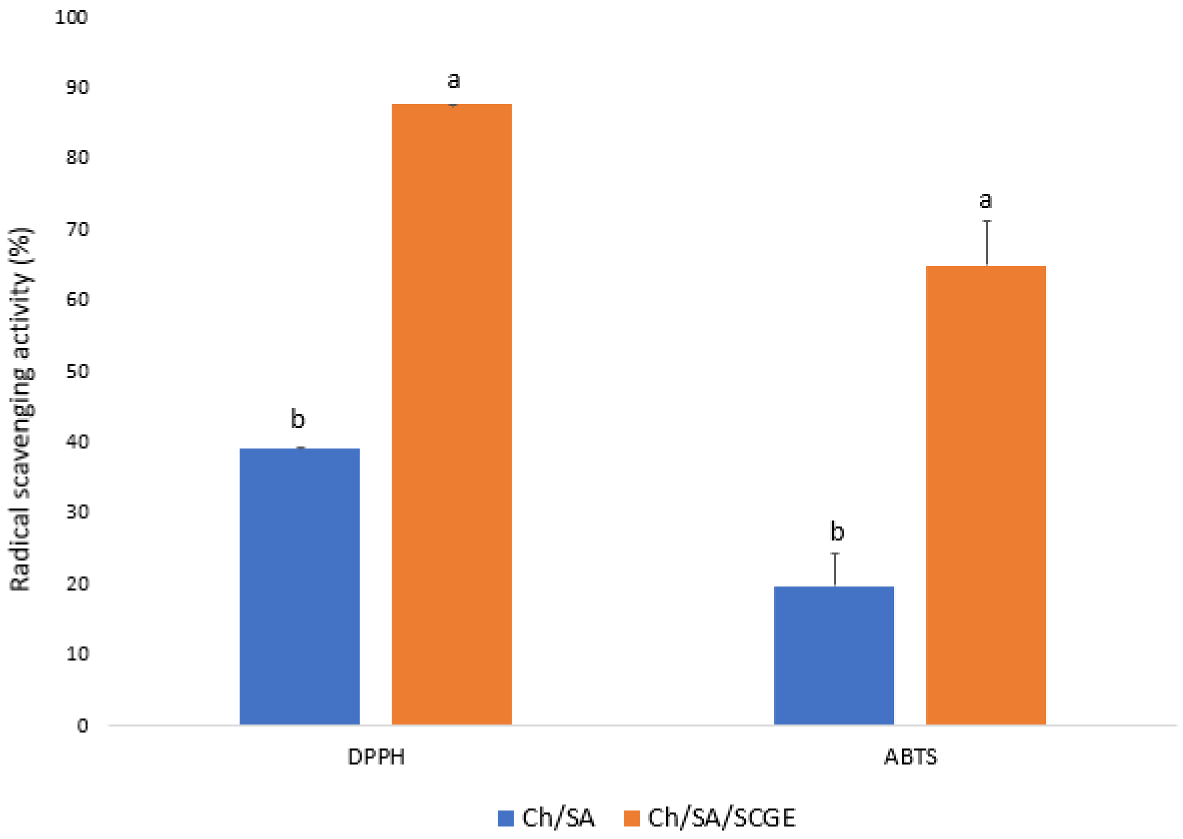

Figure S1. Antioxidant activities (DPPH and ABTS Assay)

Table S1. Levels of independent variables used in the central composite design (CCD).

Independent variables	Levels		
	Low (-1)	Centre (0)	High (+)
SCGE (% v/v)	6	8	10
Sodium Alginate (% w/v)	1	1.5	2
Chitosan (% w/v)	1	1.5	2
Glycerol (% v/v)	1	1.5	2

Table S2. Assignments of the characteristic bands of the SCGE, SA, and Ch.

**Source**	**Wavenumber (cm^{-1})**	**Assignment**
**SCGE**	3301	O-H stretching
	2927	C-H stretching vibration
	1696 and 1637	C = O and C = C stretching vibrations
	1594	C-N stretching vibration
	1368	COO– symmetric stretching vibration
	1029	C-O-C groups
	761	C-O groups
**SA**	3260	O-H stretching
	2933	C-H stretching
	1601 and 1407	symmetric and symmetric stretching vibration of the COO−
	1316 and 1085	C-O stretching
	1024	C-O-C groups
	930	uronic acid functional group
	856	mannuronic acid functional group
	3285	O-H stretching
**Ch**	2920 and 2872	C-H stretch vibrations
	1642	C=O stretching (amide I)
	1558	N−H bending (amide II)
	1410	CH2 bending
	1381	CH3 symmetrical deformation
	1152 and 1027	C-O bond stretching
	882	C-C stretch vibrations
